# Editorial on the Special Issue: “Multidrug Combinations”

**DOI:** 10.3390/biom12060812

**Published:** 2022-06-09

**Authors:** Nuno Vale

**Affiliations:** 1 OncoPharma Research Group, Center for Health Technology and Services Research (CINTESIS), Rua Dr. Plácido da Costa, 4200-450 Porto, Portugal; nunovale@med.up.pt; Tel.: +351-220426537; 2 Department of Community Medicine, Information and Health Decision Sciences (MEDCIDS), Faculty of Medicine, University of Porto, 4200-450 Porto, Portugal; 3 CINTESIS@RISE, Faculty of Medicine, University of Porto, Al. Prof. Hernâni Monteiro, 4200-319 Porto, Portugal

Biological organisms are composed of complex subsystems that interact dynamically at different levels, with functions that are complemented to avoid system malfunctions. When a disease develops, it means that these subsystems are not working properly and the appropriate treatment should focus on the multi-faced challenges of the disease, rather than a single aspect of the disease [1]. This is precisely the dynamic that occurs with cancer. Current cancer therapy includes a variety of strategies that can comprise only one type of treatment, or a combination of treatments such as surgery with chemotherapy and/or radiation therapy, immunotherapy, among others [2]. Generally, combination therapy often has superior effects compared to monotherapy [3,4]. The treatment plan is usually defined based on the type of cancer, tumor stage and patient characteristics. Despite the wide availability of treatments for cancer therapy, chemotherapy still plays a major key role in the treatment of this disease. Pharmaceutical development, applied biomedicine, and precision medicine can all benefit from pharmacological drug combination systems. In order to realize the importance of drug combination systems, the focus must be kept on oncology.

With the recent advances in omics and cell biology, the understanding of cancer as a complex disease composed of interconnected pathways has increased the interest in the use of repurposed drugs and combination therapies in oncotherapy [5]. Repurposed drugs are compounds that are already approved by FDA and that are available on the market. Drug repurposing implies the discovery of novel uses for the drug rather than its original indication [6]. Using repurposed drugs is advantageous as these agents have well-established pharmacokinetic, pharmacodynamic and toxicological profiles, making it easy for their approval for novel indications [7]. On the other hand, drug combination is another promising strategy that has been used in several areas such as cancer [8], asthma [9], AIDS [10], etc. This strategy exploits the susceptibility of different molecular pathways involved in the genesis of a certain disease to the simultaneous action of different drugs, trying to improve the efficacy of the treatment, decrease cytotoxicity to normal cells and reduce the development of drug resistance [3]. When combining two or more drugs, the main objective is to achieve interaction effects, i.e., in providing evidence of significant superiority of the combination compared to each drug individually [11]. The clinical development of combination therapies is of particular interest, mainly during the selection of drug dosages, where a vast range of possible combination doses exists [12].

Over the years, research regarding the use of combined therapies has resulted in several theoretical and experimental manuscripts that usually describe the drug interaction effects as synergistic or antagonistic, which represent, respectively, greater, or lesser effects for drugs in combination than the simple additive effect expected from the knowledge of the effects of each drug individually [11]. Nevertheless, defining additivity is not as simple as it may seem and, throughout the years, several authors have proposed different formal definitions and approaches to apply this concept in clinical practice [11,13,14,15].

Different reference models are available for the study of combination effects. The most common reference models for drug combinations can be categorized as effect-based and dose–effect-based [11]. The first models compare the effect of the combination of two drugs directly to the effects of each drug individually. Effect-based strategies encompass four main strategies: Combination Subthresholding [11], Highest Single Agent [16,17], Response Additivity [17], and the Bliss Independence model [18]. Dose–effect-based approaches consider that a comparison between drugs that have nonlinear dose–effect curves should find the appropriate concentration of each drug that results in the same effect, which depends on the dose–effect curve of each drug. This strategy includes the Loewe Additivity model [18]. A century later, after the appearance of these reference models, methods to quantify drug synergy continue to appear [13,19,20] based on these two principles. Nevertheless, the models for drug combinations still have some limitations in their clinical application and research regarding this topic continues to be of particular importance. We have no doubt that the next advances in medicine may result from synergistic studies or from interaction between therapeutic regimens or simply combinations of drugs.
